# Mapping the sex determination locus in the Atlantic halibut (*Hippoglossus hippoglossus*) using RAD sequencing

**DOI:** 10.1186/1471-2164-14-566

**Published:** 2013-08-20

**Authors:** Christos Palaiokostas, Michaël Bekaert, Andrew Davie, Mairi E Cowan, Münevver Oral, John B Taggart, Karim Gharbi, Brendan J McAndrew, David J Penman, Hervé Migaud

**Affiliations:** 1 Institute of Aquaculture, School of Natural Sciences, University of Stirling, Stirling, Scotland, FK9 4LA, UK; 2 The GenePool, School of Biological Sciences, University of Edinburgh, Edinburgh, Scotland, EH9 3JT, UK

**Keywords:** Hippoglossus hippoglossus, Sex determination, Monosex, QTL mapping, RAD-seq, Aquaculture

## Abstract

**Background:**

Atlantic halibut (*Hippoglossus hippoglossus*) is a high-value, niche market species for cold-water marine aquaculture. Production of monosex female stocks is desirable in commercial production since females grow faster and mature later than males. Understanding the sex determination mechanism and developing sex-associated markers will shorten the time for the development of monosex female production, thus decreasing the costs of farming.

**Results:**

Halibut juveniles were masculinised with 17 α-methyldihydrotestosterone (MDHT) and grown to maturity. Progeny groups from four treated males were reared and sexed. Two of these groups (n = 26 and 70) consisted of only females, while the other two (n = 30 and 71) contained balanced sex ratios (50% and 48% females respectively). DNA from parents and offspring from the two mixed-sex families were used as a template for Restriction-site Associated DNA (RAD) sequencing. The 648 million raw reads produced 90,105 unique RAD-tags. A linkage map was constructed based on 5703 Single Nucleotide Polymorphism (SNP) markers and 7 microsatellites consisting of 24 linkage groups, which corresponds to the number of chromosome pairs in this species. A major sex determining locus was mapped to linkage group 13 in both families. Assays for 10 SNPs with significant association with phenotypic sex were tested in both population data and in 3 additional families. Using a variety of machine-learning algorithms 97% correct classification could be obtained with the 3% of errors being phenotypic males predicted to be females.

**Conclusion:**

Altogether our findings support the hypothesis that the Atlantic halibut has an XX/XY sex determination system. Assays are described for sex-associated DNA markers developed from the RAD sequencing analysis to fast track progeny testing and implement monosex female halibut production for an immediate improvement in productivity. These should also help to speed up the inclusion of neomales derived from many families to maintain a larger effective population size and ensure long-term improvement through selective breeding.

## Background

The mechanisms of sex determination in animals are remarkably diverse. Gonochoristic animals show genetic and/or environmental sex-determining mechanisms. Genetic sex-determining systems can be either chromosomal, and involve a master sex-determining gene/region on a sex chromosome, or can be polygenic and involve several genes/regions on multiple chromosomes. In most fish species with XX/XY or ZZ/ZW mechanism, the sex chromosomes do not show clear differences in length or gene content [1]. Several fish sex determining genes have been isolated from species with XX/XY mechanisms: *DMY/dmrt1bY* in *Oryzias latipes* (medaka) [2]; *Gsdf(Y)* in *Oryzias luzonensis* (Luzon ricefish) [3]; *amhy* in *Odontesthes hatcheri* (Patagonian pejerrey) [4]; *Amhr2* in *Takifugu rubripes* (tiger pufferfish) [5]; and *sdY* in *Oncorhynchus mykiss* (rainbow trout) [6]. In environmental sex-determining systems, the environment plays a decisive role, such as temperature in turtles, alligators and fish [1,7,8]. Both systems can interact in some species such as in *O. latipes*, which has an XX/XY genetic system, where high temperatures can cause female-to-male sex reversal [9-11]. Additionally, autosomal loci can also contribute to sex determination in many species [12]. Overall, the understanding of sex determination systems in fish has direct commercial applications, given the strong sexual dimorphism exhibited in a wide variety of aquaculture fish species for a range of commercially important traits like growth or age at maturity.

*Hippoglossus hippoglossus* (Atlantic halibut) has been a high-value species for cold-water marine aquaculture for several decades in Northern Europe and America, although production has been limited by a series of bottlenecks. Among these, sexual dimorphism in growth, with males maturing earlier and growing significantly slower than females, reduces productivity and profitability of the sector. Females can reach market size (3-5 Kg) at around 36 months while males require at least an extra year, making the production of all-female stocks particularly appealing for the aquaculture industry [13,14].

Flatfish (order Pleuronectiformes) show a range of sex-determining mechanisms, including XX/XY and ZZ/ZW, with significant effects of environmental factors, principally temperature, in some species [15]. Meiotic gynogenetic *H. hippoglossus* were all-female, suggesting an XX/XY sex-determining system [16]. Temperature has not been shown to have an effect on *H. hippoglossus* sex ratio [17]. Gonadal sex differentiation can be manipulated through in-feed synthetic steroid treatments (*e.g.*, 17 α-methylhydrotestosterone, MDHT or 17 β-estradiol [18]) or aromatase inhibitor treatments (*e.g.*, Fadrozole [14]). However, direct sex reversal is not a commercially acceptable means to alter sex ratios in food fish within the EU [19]. Thus indirect sex reversal is required, whereby masculinised genotypic females (XX neomales) are crossed to normal females (XX) to produce genetically all-female progeny, a process which has yet to be proven in *H. hippoglossus*. The crux of successful indirect sex reversal is the non-lethal identification of the neomales. Currently the main technique for such verification is progeny testing of treated animals which is time consuming and costly, taking at least four or five years due to the timing of puberty in halibut (reached after three years). Direct genetic sexing, instead of progeny testing, would be preferable using non-lethal and cheap genotyping techniques. This is only likely to be possible in simple cases of male or female heterogamety. Sex-specific genomic sequences are only available in a limited number of aquaculture species [20]. Although a genetic linkage map based on microsatellites and amplified fragment length polymorphism (AFLP) is available for *H. hippoglossus*[21], this does not contain any information about sex-determination. Restriction associated DNA (RAD) sequencing is a powerful technique for generation of high-density linkage maps and conducting quantitative trait locus (QTL) analysis [22,23] including the mapping of sex-determining loci in fish [24].

The aim of the current research was to demonstrate that indirect sex reversal was possible and thereafter to develop sex-associated markers through RAD-sequencing. An in-feed MDHT treatment was given to weaned halibut juveniles during the labile period, which resulted in 97% phenotypic males. A sub-population of these treated fish was then reared to maturity and from this stock, two neomales and two normal males were verified by progeny testing, a process that took four years to complete. The sex-determining locus was mapped to the end of the linkage group 13, in the two mixed sex families from the sex reversal study, using polymorphic Single Nucleotide Polymorphisms (SNP). A combination of four markers predicted sex with 97% accuracy in any individual fish, from a panel of progeny and broodstock. Synteny analysis showed that DNA sequences containing Atlantic halibut sex-associated SNPs were consistently clustered in several other fish genomes. These results suggested that sex determination in *H. hippoglossus* is likely to be monogenic (XX/XY) and localised within a 3.2 cM window on linkage group 13.

## Results

### Hormonal sex reversal and neomale verification

The control group exhibited a sex ratio not significantly different from 1:1 (52% ♂; 48% ♀), whereas 97% of the group treated for six weeks (5 ppm) and 70% of the group treated for three weeks (10 ppm) were confirmed as phenotypic males (Table 1). Both in-feed treatments significantly altered the natural sex ratio in favour of males. Of the seven putative neomales that were progeny tested, only four crosses produced enough survivors at the age of sexing, at approximately one year of age. From these four crosses, two gave 100% female progeny (Families A and D; Table 1) while the other two gave balanced sex ratios (Families B and C; Table 1).

**Table 1:** Sex ratios in hormonal masculinisation trial (control, 5 ppm and 10 ppm MDHT) and progeny testing (families A-D from the four males from the 5 ppm MDHT group)

	**Control**	**5 ppm**	**10 ppm**	**A**	**B**	**C**	**D**
**N**	77	77	76	26	30	71	70
**Male (Obs. / Exp.)**	40 / 38.5	75 / 38.5	53 / 38	0 / 13	15 / 15	32 / 30.5	0 / 35
**Female (Obs. / Exp.)**	37 / 38.5	2 / 38.5	23 / 38	26 / 13	15 / 15	29 / 30.5	70 / 35
**χ**^**2**^	0.12	69.21	11.8	26	0	0.15	70
***P*-value**	0.824	< 0.001	< 0.001	< 0.001	1	0.797	< 0.001
**Sex Ratio**	52% male	97% male	70% male	100% female	50% female	48% female	100% female

Obs.; observed; Exp.: expected under H_0_ hypothesis (1:1 ratio).

### RAD sequencing

**Figure 1:**
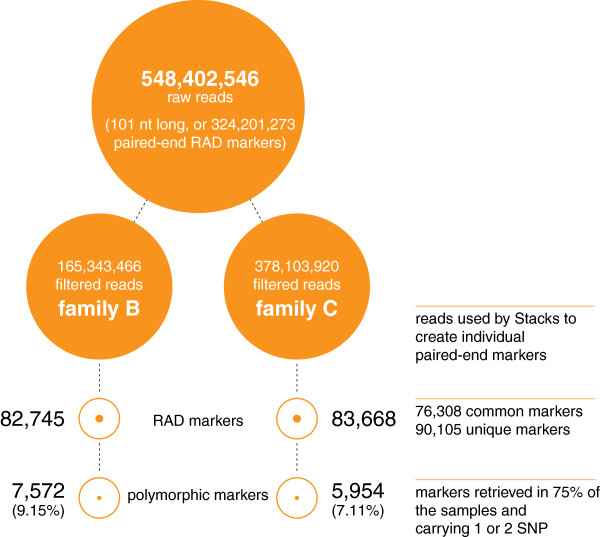
**Sequencing and RAD-tag summary.** Detail of the number of reads before and after filters (orange disk) followed by the reconstructed number of RAD markers and polymorphic RAD markers (orange circles).

Two crosses with 62 (Family C) and 28 offspring (Family B) and their parents (including a common female) were analysed (Additional file 1). The DNA samples were barcoded, pooled and sequenced in two lanes of an Illuminia HiSeq 2000 sequencer (see Methods). In total, 648,402,546 raw reads (101 bases long) were produced (or 324,201,273 paired-ended reads: NCBI BioProject accession number SRP016043). After removing low quality sequences (quality score under 30), ambiguous barcodes and orphaned paired-end reads, 81.24% of the raw reads were retained (526,783,920 reads). The Stacks package [25] was then used to make a *de-novo* assembly of the sampled loci from each individual: 82,745 and 83,668 RAD-tags were retrieved for Families B and C respectively, covering 90,105 RAD-tags in total including 76,308 of these shared between the two families (Figure 1). The number of reads and RAD-tags for each sample are reported in Additional file 1.

### Genetic map

**Figure 2:**
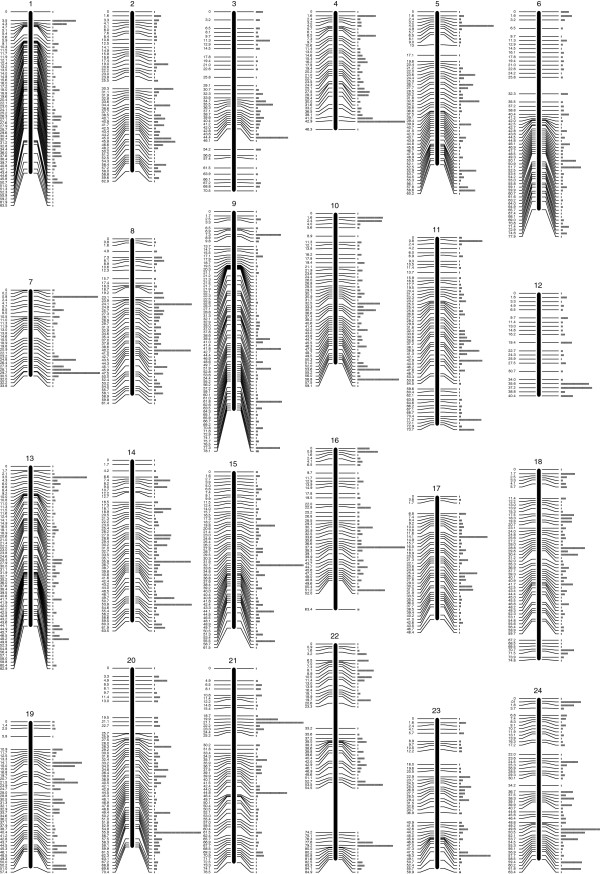
**Genetic linkage map.** Map with linkage group assignment determined using syntenic markers with previously published *H. hippoglossus* maps. The positions on the left side of the chromosomes are in cM. The rectangles on the right hand side represent the number of markers at this position. Detailed data is provided in the Additional file 2.

In order to maximise the number of informative markers and minimise the amount of missing or erroneous data, we used only paired-end RAD-tags retrieved in at least 75% of the samples in each family, and carrying one or two SNPs. 7572 and 5954 RAD-tags were retained for the families B, and C respectively (Figure 1). Since Family B had only 28 offspring, the genetic map was constructed with the Family C data only (62 offspring). The map consists of 5703 SNPs and 7 microsatellites (used initially for parentage assignment) in 24 linkage groups (LG) and spanning 1514 cM (Figure 2; Additional file 2). 4049 of the above SNPs were common in the two families and were used to incorporate the data from Family B into a joint linkage map. Sex-specific genetic maps were constructed, with the female-specific map spanning 1496 cM and the male-specific map spanning 1378 cM (Table 2).

**Table 2:** ***H. hippoglossus *genetic map**

**Linkage group**	**No. of markers**	**Size (cM)**	**Female (cM)**	**Male (cM)**
1	215	63.5	60.0	63.5
2	240	62.9	62.9	58.0
3	177	70.5	67.2	38.2
4	229	46.3	44.7	28.4
5	241	60.2	58.6	58.6
6	196	77.9	74.6	37.5
7	230	33.8	32.0	33.8
8	268	61.4	61.4	61.4
9	265	78.1	78.1	78.1
10	293	59.1	57.5	57.5
11	246	73.7	73.7	73.7
12	186	69.2	40.4	64.3
13	222	62.9	62.9	60.8
14	318	63.5	61.9	63.5
15	269	61.5	61.5	59.9
16	237	63.4	63.4	63.4
17	174	48.4	48.4	48.4
18	257	74.8	73.1	74.8
19	250	57.4	57.4	51.0
20	269	70.4	70.4	44.7
21	260	76.5	76.5	66.0
22	250	84.9	84.9	81.7
23	169	58.9	57.3	49.1
24	303	63.4	63.4	61.8
Size (cM)		1514	1496	1378.1
Markers		5710	3858	3412

Three linkage maps were constructed using all 5710 segregating polymorphic markers from males, females or both.

### QTL-association mapping

**Figure 3:**
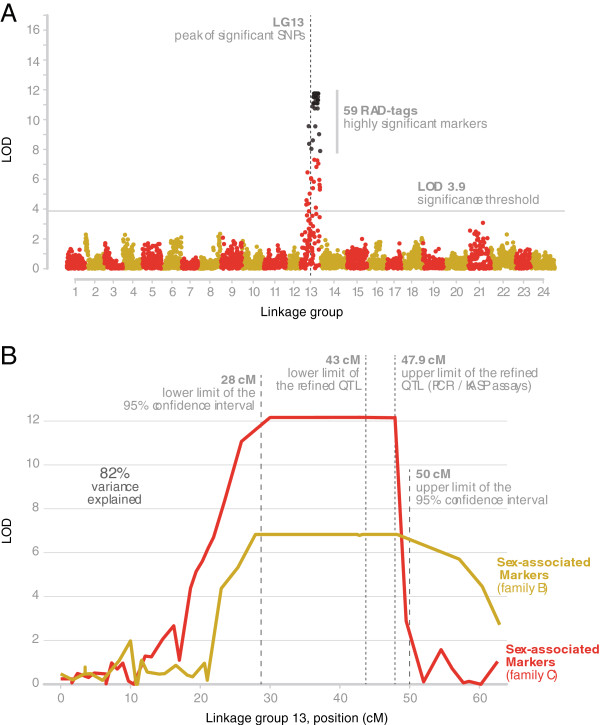
**Results from QTL-Analysis. (A)** Association results for genotyped SNPs. SNPs with *p*-values achieving genome-wide significance (*P* < 7.2 × 10-8) are shown in black. **(B)** Regional analysis of the QTL on LG 13. Plot of the LOD score (sex-association QTL search) along the linkage group 13 for family **B** and **C**.

The results from the single-QTL model for binary traits provided evidence for the existence of a major QTL in LG 13 for both families in the male informative dataset (Figure 3A). The highest logarithm of odds (LOD) scores for Families B and C were 6.83 and 12.17 respectively (Figure 3B). The genome-wide thresholds (α = 0.01) were calculated from permutation tests (10,000 permutations) to be 3.25 and 3.90 respectively. The highest LOD scores were observed in the region between 30 cM and 48 cM in LG 13 for both families. Even though models that take into account the existence of a major QTL (as in this study) or ones that test for existence of multiple QTLs simultaneously reduce the residual variation (providing this way higher power in the analysis for detecting additional QTLs at least of modest effect), no additional QTLs were detected. The calculated 95% Bayesian Density Intervals for the QTL location spanned a region of 22 cM (28-50 cM in LG 13), while the 1.5-LOD support interval spanned a region of 28 cM (23-51 cM in LG 13). The variance explained by the QTL was around 82%. There were no significant QTL in the female informative dataset. The Polymorphism Information Content (PIC) of the markers in LG 13 ranged from 0.64 to 0.99. The joint QTL analysis of the two families showed a maximum at 46 cM with a Likelihood Ratio (LR) of 96 (Figure 3B). The chromosome-wide threshold calculated from permutation tests (10,000 permutations) was LR = 19. The association analysis identified 38 SNPs being strongly associated (*P* < 0.001) with sex in the two families. Models scanning simultaneously for the existence of two QTL or multiple QTL did not reveal any extra QTL.

### Verification of SNP sex association and sex prediction

**Figure 4:**
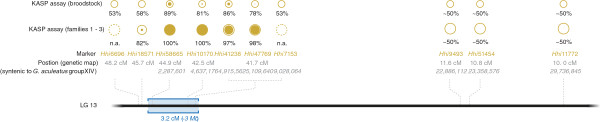
**KASP assay and fine gene mapping on LG 13.** Details of the 10 markers tested by KASP assay. From bottom to top: Location of the 10 markers (in the genetic map in cM and syntenic loci on the *G. aculeatus*, three-spined stickleback, group XIV in bp); KASP assay results. The outer circle diameters for the KASP assay results are proportional to the number of alleles tested. The inner (solid) disks represent the marker association with the phenotypic sex. Detailed data is provided in Additional file 4. When no informative polymorphism was found, “n.a.” is specified.

Sex association of 10 SNP markers (Additional file 3) selected from LG 13 was investigated using allele specific endpoint-genotyping assays (Figure 4; Additional file 3). The *P*-value thresholds (α = 0.05) after taking into account multiple testing (for the 10 SNP markers that were tested) according to the permutations and the Bonferroni correction tests were found to be 0.0054 and 0.0050 respectively. *Hhi*58665 (*P* = 3.05 × 10^-19^), *Hhi*10170 (*P* = 3.05 × 10^-19^), *Hhi*41238 (*P* = 2.45 × 10^-16^) and *Hhi*47769 (*P* = 8.96 × 10^-17^) showed the highest association with sex, over 97% in the three test families (n = 10 ♀:10 ♂ in Families 1 & 2; 9 ♀:9 ♂ in Family 3; total 58). In each case the parents were a heterozygous ♂ and a homozygous ♀ for each marker. Furthermore when tested in 36 wild sourced broodstock from locations across the species native range (Additional file 4), the same four markers individually showed between 78% and 89% association with sex (*P* < 0.001), with SNP *Hhi*58665 showing the highest association (*P* = 6.39 × 10^-7^). These four markers were located within a 3.2 cM region of LG 13.

**Figure 5:**
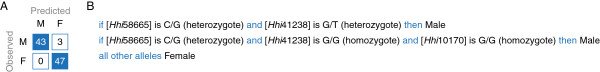
**Combined marker sex prediction. (A)** Confusion matrix of the JRip rules. Blue cells are correct predictions; white cells are the erroneous predictions. Overall the predictions are 97% accurate. **(B)** JRip rules based on the alleles detected using the KASP assays.

The combined prediction power of these four markers was tested on the 36 broodstock and 58 progeny using the JRip classifier, as the derived rules have straightforward interpretation. The combined prediction is based on two rules using three markers (Figure 5) and produced 97% corrected classification with the 3% of errors being phenotypic males predicted to be females. The male prediction precision is 1 (recall of 0.935) and the female prediction precision is 0.94 (recall of 1).

Within the 18 tested male broodstock, two had “female” genotypes for all four of these markers (one of the 58 progeny also had male phenotype but female genotype). One of these two broodstock had previously been crossed with four females and had produced only female offspring (between 3 and 14 individuals per family, total 27). The phenotypic sex of these 27 offspring was verified by post-mortem examination three years post-fertilisation.

### Synteny searches

We selected the 59 markers within the 95% confidence interval around the LOD score peak and mapped them onto the genomes of related species to identify syntenic regions. We performed this search against *Danio rerio* (zebrafish), *Gadus morhua* (Atlantic cod), *Gasterosteus aculeatus* (three-spined stickleback), *Latimeria chalumnae* (West Indian ocean coelacanth), *Oreochromis niloticus* (Nile tilapia), *Oryzias latipes* (medaka), *Takifugu rubripes* (tiger pufferfish) and *Tetraodon nigroviridis* (spotted green pufferfish) genomes. 33 markers had unique hits across at least five out of eight species (Additional file 5). *G. aculeatus* and *O. niloticus* show the highest level of synteny with *H. hippoglossus* and each other (Additional file 5). The order of the markers selected for SNP genotyping in the regions point toward one 3.2 Mb region embedding more than 60 annotated genes (See direct links to the Ensembl 68 in Additional file 5). No genes associated with sexual differentiation or determination were identified in this region.

## Discussion

*H. hippoglossus* is a species of increasing commercial interest for cold-water marine aquaculture. However one of the main limitations to profitable culture of the species is the sexual dimorphism in age at maturation related to gender specific growth performance [13,14]. To address this bottleneck, the current study demonstrates, for the first time in this species, that indirect monosex female production is possible for commercial *H. hippoglossus* aquaculture. While having strong commercial application, this research also had the fundamental aim to investigate the genomic regulation of sex determination in the species through state-of-the-art high-throughput sequencing methodologies.

RAD-tag sequencing has recently been used with a number of different fish species since the technology became available in 2008. One of the aims of the Baird et al. [23] study, which first validated the technique in fish, was to fine map QTLs in *G. aculeatus*. A number of different restriction-digest methodologies already existed, using high-throughput sequencers. However, what sets the RAD-tag methodology apart is the fact that it combines control over the fragments that result from the digestion with deep sequencing across individuals, making the identified SNP reproducible [26]. This makes the RAD platform very efficient for constructing genetic maps and QTL studies.

In the present study, a genetic map of 5703 SNPs and 7 microsatellites spanning 1514 cM was constructed. To our knowledge this is the first dense genetic map incorporating SNPs in any flatfish species. The map has 24 linkage groups, corresponding to the number of chromosome pairs in *H. hippoglossus*[27]. In a similar study, Amores et al. [28] constructed a genetic map for *Lepisosteus oculatus* (spotted gar) consisting of 8406 SNPs. The above map was used to prove that *L. oculatus* diverged from teleosts before the Teleost genome duplication. Genetic maps of more than 4500 SNPs using RAD-seq were also constructed in *O. mykiss* and in *D. rerio*[24,29].

The high LOD (> 10), which was used to assign the genetic markers in linkage groups in our study, ensures that the map is of high quality. However, it must be acknowledged that even though the assignment of markers in linkage groups is robust, none of the available algorithms used for ordering markers provides an accurate positioning of closely spaced markers due to the relatively low number of meioses represented in our sample size. In a species like *H. hippoglossus* with no sequenced genome available, a genetic map is an invaluable tool for mapping any trait of interest in a QTL study. Apart from mapping QTL, the identified SNP of the genetic map can be used to construct a genomic relationship matrix, which can replace the relationship matrix inferred by pedigree for calculating breeding values. This would improve accuracy of estimated breeding values (EBV) under Best Linear Unbiased Prediction (BLUP) methodology in a breeding program [30]. The improvement in accuracy is due to the fact that the genomic relationship matrix accounts for the random segregation of chromosome segments at meiosis between siblings.

In this study we associated mapped RAD-tag markers to sex determination. A major QTL involved in sex determination was identified in LG 13 in both families (LOD = 12.16 and 6.83 in Family B and C respectively). The location of the above QTL spans a region of around 22 cM. This region should contain one or more genes responsible for sex determination in *H. hippoglossus*. The reduced recombination in this region resulted in an almost flat likelihood surface for this region. Genome regions with reduced recombination are a common characteristic of sex chromosomes. In a similar study by Anderson et al. [24] where the objective was to identify QTLs involved in sex determination in zebrafish using RAD-tag, a region in chromosome 4 spanning more than 20 cM showed reduced recombination. In general suppression of recombination keeps together genes (or alleles) with functions that are advantageous for one sex and avoids their transfer to the other sex chromosome, where they might have negative effects on the opposite sex [31].

Our data support the hypothesis that the *H. hippoglossus* has an XX/XY sex determination system. Among flatfish species, *Paralichthys olivaceus* (Bastard halibut) has also been shown to possess an XX/XY system [32], although temperature also influences sex ratio. On the other hand other closely related species, in which sex associated genetic markers have been identified, such as *Hippoglossus stenolepis* (Pacific halibut) [33], *Verasper variegates* (spotted halibut) [34], *Scophthalmus maximus* (turbot) [35] and *Cynoglossus semilaevis* (half-smooth tongue sole) [36] were all shown to have a ZZ/ZW sex determination system. Unusually in this group, *C. semilaevis* has differentiated W and Z chromosomes [36].

Validating the results of the QTL-Association Analysis is of the utmost importance. The fact that the sex-associated SNPs showed strong association when tested in a wider panel of three families and 36 wild broodstock provides clear evidence that those markers are in strong linkage disequilibrium with the sex-determining gene(s). Marker-assisted selection (MAS) could be conducted using these SNPs, providing a valuable tool towards more efficient production of all-female stocks for the aquaculture industry. In the current study it took four years from initiation of sex reversal treatment to completion of progeny testing for neomale identification with guaranteed all-female production from the following year. By employing MAS however it would be possible to confirm sex associated genotype from a non-destructive biopsy sample in hormonally-treated fish within 6-12 months of treatment, allowing neomales to be isolated and used from first maturation at three-four years post-treatment. SNPs *Hhi*58665, *Hhi*10170, *Hhi*41238 and *Hhi*47769 are the strongest candidates for MAS since they correctly assign sex in more than 97% of the screened individuals. They span a narrow region of 3.2 cM. Genotyping a larger population for the SNPs in this region would allow fine mapping of the sex-determining locus. Other genetic factors involved in sex determination might also be involved.

The application of this technology will enable the industry to include a greater number of neomales from a wider genetic base to be included in future breeding programmes without the reduction in effective population size (Ne) associated with the use of a small number of neomales from these initial sex-reversed families. Limited examples exist of practical application of MAS in breeding programmes in aquaculture. A Y-specific DNA marker was used to assist in the development of monosex female culture in *Oncorhynchus tshawytscha* (Chinook salmon) [37]. More recently, MAS has been apply to a QTL for Infectious Pancreatic Necrosis Virus resistance in *Salmo salar* (Atlantic salmon): initially microsatellite markers were used, and more recently SNPs derived from RAD sequencing have been added [38,39].

## Conclusions

Overall this work has demonstrated that all-female halibut production is commercially possible using indirect monosex production techniques. This in itself confirms that *H. hippoglossus* has an XX/XY sex determination system. RAD-tag sequencing produced 90,105 unique loci, and a single sex determination locus was mapped to LG 13. A further set of 4 markers that were present only or predominantly in DNA from male fish was isolated from two families and validated in a wider population screening, opening the possibility of MAS for sex in the species. Synteny analysis showed that DNA sequences containing *H. hippoglossus* sex-associated SNPs were consistently clustered in several other genomes, which provides a new focus for research into the sex determination mechanism in this species.

## Methods

### Ethics statement

All working procedures complied with the United Kingdom Animals (Scientific Procedures) Act 1986 and were approved by the ethics committee of the University of Stirling.

### Hormonal sex reversal

Weaned mixed-sex halibut larvae (mean total length of 40.1 ± 0.2 mm, mean wet weight of 0.5 ± 0.01 g) produced in the 2007 spawning season, were obtained from a commercial halibut hatchery and transferred to the Machrihanish Marine Environmental Research Laboratory (55.424°N, 5.749°W) for hormonal treatment. Three in-feed treatments were tested in duplicate: a) 6 weeks steroid free diet (control), b) 6 weeks MDHT in-feed (5 ppm) [18] and c) 3 weeks MDHT in-feed (10 ppm) followed by 3 weeks steroid-free diet. Food was provided in excess by automated feeders into the tanks every 12 minutes throughout a 24-hour period. Feed, based on a commercial diet (Low Energy Marine Larval diet, EWOS, West Lothian, UK), was mixed with an ethanol solution containing the appropriate dose of MDHT (Sigma-Aldrich Co Ltd, Poole, UK) and then dried in an extraction fume hood. Following treatment and once the fish had reached a mean weight of 28.4 ± 0.4 g, replicate treatment groups were identified by a coded subcutaneous dye mark and then reared communally. At approximately 1 year post fertilisation a total of 80 individuals per treatment (40 per replicate) were sacrificed and fixed in 4% neutrally buffered formalin for histological determination of phenotypic sex. Sex ratios were compared to the expected 1:1 and were evaluated statistically using a Chi-square test, (*P* < 0.05, χ^2^ = 3.84, df = 1).

### Neomale verification by progeny testing

At a mean size of 180.8 ± 3.1 g, 60 control fish (30 per replicate) and 150 fish from the 5 ppm treatment (75 per replicate) were tagged with a passive integrated transponder tag (Fish Eagle Co., Lechlade, UK). Fish were then reared communally until first maturity in spring 2010. In March 2010, crosses were performed between 7 males from the hormone-treated population and normal female broodstock. Fertilisation was confirmed in each cross by microscopic examination of blastomere development. Eggs from each cross were maintained in isolation using standard commercial rearing methodologies. Sufficient progeny from only four of these males survived through yolk sac absorption, live feeding and weaning. These four families were reared in isolation at a commercial halibut hatchery until phenotypic sex ratio could be assessed in February-March 2011, once fish reached a suitable size (over 50 g) for histological sexing of the gonads. A total of 30 (family A & B) or 70 (family C & D) individuals/family were sacrificed for histological examination and blood was sampled for genotyping (total of 200 offspring). Sex ratios were compared to the expected 1:1 using a Chi-square test (*P* < 0.05, χ^2^ = 3.84, df = 1).

### RAD library preparation and sequencing

DNA was extracted from blood samples of the fish using the REALPure genomic DNA extraction kit (Durviz S.L.) and treated with RNase to remove residual RNA from the sample. Each sample was quantified by spectrophotometry (Nanodrop) and quality assessed by agarose gel electrophoresis, and was finally diluted to a concentration of 50 ng/μL in 5 mmol/L Tris, pH 8.5. The RAD library preparation protocol followed essentially the methodology originally described in Baird et al. [23] and comprehensively detailed in Etter et al. [40], with the minor modifications described in Houston et al. [38]. The RAD-specific P1 and P2 paired-end adapters and library amplification PCR primer sequences used in this study are detailed in Baxter et al. [41].

Each sample (1.5 μg parental DNA / 0.5 μg offspring DNA) was digested at 37°C for 30 minutes with *Sbf*I (recognising the CCTGCA|GG motif) high fidelity restriction enzyme (New England Biolabs; NEB) using 6U *Sbf*I per μg genomic DNA in 1× Reaction Buffer 4 (NEB) at a final concentration of c. 1 μg DNA per 50 μL reaction volume. The reactions (75 / 25 μL final volumes for parental / offspring samples respectively) were then heat inactivated at 65°C for 20 minutes. Individual specific P1 adapters, each with a unique 5 bp barcode (Table 1), were ligated to the *Sbf*I digested DNA at 22°C for 45 minutes by adding 3.75 / 1.25 μL 100 nmol/L P1 adapter, 0.9 / 0.3 μL 100 mmol/L rATP (Promega), 1.5 / 0.5 μL 10× Reaction Buffer 2 (NEB), 0.75 / 0.25 μL T4 ligase (NEB, 2 M U/mL) and reaction volumes made up to 90 / 30 μL with nuclease-free water for each parental / offspring sample. Following heat inactivation at 65°C for 20 minutes, the ligation reactions were slowly cooled to room temperature (over 1 hour) then combined in appropriate multiplex pools (Additional file 1). Shearing (Covaris S2 sonication) and initial size selection (250-500 bp) by agarose gel separation [38] was followed by gel purification, end repair, dA overhang addition, P2 paired-end adapter ligation, library amplification, exactly as in the original RAD protocol [23,40]. A total of 150 μL of each amplified library (14 PCR cycles) was size selected (c. 300-550 bp) by gel electrophoresis [38]. Following a final gel elution step into 20 μL EB buffer (MinElute Gel Purification Kit, Qiagen), the libraries were sequenced at The GenePool Genomics Facility at the University of Edinburgh, UK, for quality control and high-throughput sequencing. Libraries were accurately quantified by qPCR (Kapa Library) and run in two lanes of an Illumina HiSeq 2000 using 100 base paired-end reads (v3 chemistry). Raw reads were process using RTA 1.12.4.2 and Casava 1.6 (Illumina). The reads were deposited at the NCBI BioProject under the accession SRP016043.

### Genotyping RAD alleles

Reads of low quality (score under 30, while the average quality score was 37), missing the restriction site or with ambiguous barcodes were discarded. Retained reads were sorted into loci and genotyped using Stacks software 0.9995 [25]. The likelihood-based SNP calling algorithm [42] implemented in Stacks evaluates each nucleotide position in every RAD-tag of all individuals, thereby differentiating true SNPs from sequencing errors. The parameters were a minimum stack depth of at least 30, a maximum of 2 mismatches allowed in a locus in an individual and up to 1 mismatch between alleles. The pair-ends were assembled using Stacks and Velvet version 1.2.08 [43] and used to separate RAD-tag sequence with or without potential SNP but belonging to separate loci (duplication products). Polymorphic RAD-tags may contain more than one SNP, but the vast majority (over 99%) showed only two allelic versions; the very small proportion of RAD-tags with more than two alleles were excluded.

### Genetic map construction

The genetic map was constructed using R/Onemap [44] and TMAP [45]. The allocation of markers in linkage groups was conducted using R/Onemap. This package uses Hidden Markov Models (HMM) algorithms for outbred species while in parallel implements the methodology described in Wu et al. [46] for calculating the most probable linkage phase. Linkage groups were formed using minimum LOD values of 10. TMAP was used to order the markers in every linkage group. By using an HMM maximum likelihood model and taking into account potential genotypic errors it reduces the tendency to erroneously derive oversized linkage groups, a phenomenon which is often observed in dense maps [45]. Map distances were calculated in centiMorgans (cM) using the Kosambi mapping function. The genetic map was drawn and aligned using Genetic-Mapper v0.3 [47].

### QTL association mapping

The QTL analysis was performed using three different suites of programmes: R/qtl [48], GridQTL [49] and QtlMap [50]. In the case of R/qtl the genotypes the two families were analysed separately. The analysis was performed considering the cross as a ‘pseudo’ backcross, effectively analysing male and female informative markers separately. The model used for the analysis was based on Interval Mapping. The phenotype was considered a binary trait (0 for females and 1 for males). The algorithm used considers the phenotype to follow a mixture of Bernoulli distributions and uses a form of the EM algorithm for obtaining maximum likelihood estimates [48]. Two-way and multiple QTL models were also run with this package. Approximate Bayesian and 1.5-LOD 95% density and confidence intervals were calculated respectively. An approximate estimate of the phenotypic variance explained by the QTL was obtained from the following equation: 1-10^-2LOD/n^. While the estimated variance may be reasonable for additive QTL, problems can be caused in the case of linked QTL [48]. The GridQTL software was used to estimate the polymorphism information content across the genetic map. QTLMap was used for performing a joint QTL Analysis of the two families. The phenotype was considered as discrete and the model used was a Mixture Linkage Analysis model, accounting for heteroskedasticity. An Association Analysis was performed for the two families using R/GenABEL [51] in order to identify SNPs associated with sex. The SNP data were tested for association using the fast score test for association [52]. In all the above analysis genome-wide significance thresholds were calculated by permutation tests (10,000 permutations) in order to correct for multiple testing.

### Verification of SNP sex association

Marker sex association was tested using 10 competitive fluorescent, allele specific endpoint-genotyping assays (KASP v4.0, LGC genomics) based on SNPs that were commonly found in the two mapping families to span the region of highest association with sex (*Hhi6696, Hhi7153, Hhi9493, Hhi10170, Hhi11772, Hhi18571, Hhi41238, Hhi47769, Hhi51454, Hhi58665*, NCBI dbSNP accession 749737483, 749737484, 749737485, 749737486, 749737487, 749737488, 749737489, 749737490, 749737491 and 749737492 respectively; Additional file 3). SNP-specific primer sets were designed by LGC genomics (Additional file 3). Each genotyping assay was run in an 8 μl volume containing approximately 40 ng of target gDNA incorporated with a proprietary reaction mix in accordance with the manufacturer’s guidelines. All assays were run using the same touchdown thermal cycling programme as follows: 94°C for 15 minutes followed by 10 cycles of 94°C for 20 seconds melt, 65-57°C for 1 minute anneal and extension (decreasing of 0.8°C per cycle) followed by 26 cycles of 94°C for 20 seconds melt, 57°C for 1 minute anneal and extension. There was one exception, SNP *Hhi*58665, for which the extension time was extended to 2 minutes. All assays were run in a Biometra TGradient thermal cycler (Biometra GmbH, Goettingen, Germany). Thereafter assays results were read at 25°C using an endpoint genotyping programme in a Techne Quantica qPCR thermal cycler (Bibby Scientific Ltd, Stone, UK) in which unknown genotypes were assigned based on fluorescent output in comparison to non-template control wells containing DNA/RNA free H_2_O. All 10 SNP assays were tested in 58 offspring from three halibut families produced in the commercial halibut hatchery, which were independent from the initial mapping families, and in 36 independent broodstock halibut (18 ♀:18 ♂) originating from the Shetland Isles, Iceland and possibly the Faroe Islands. An association analysis was performed using R/SNPassoc [53]. In the case of family data, association was tested both in separate families and across all families together. A Bernoulli generalised linear model was applied in order to test the magnitude of association between the SNP genotypes and phenotypic sex using this package (function *association*). Both the Bonferroni and permutation tests (10,000 permutations) were used in order to correct for multiple testing.

### Sex prediction

The KASP allele type of all markers for each individual tested along with their sex were entered into the WEKA package [54], which contains a variety of machine-learning algorithms, including JRip, an optimised rule learning algorithm. This classifier implements a propositional rule learner, Repeated Incremental Pruning to Produce Error Reduction (RIPPER), which was proposed by Cohen [55] as an optimised version of IREP. JRip builds additive rules based on the allele type of the markers. JRip then classifies each individual into a particular predicted sex based on the allele type of the markers for each individual. Permutatively, one individual was removed from the training set, and subsequently the algorithm then assigns its sex. The set of rules was stable between permutations (Figure 5).

### Synteny searches

*D. rerio*, *G. morhua*, *G. aculeatus*, *L. chalumnae*, *O. niloticus*, *O. latipes*, *T. rubripes* and *T. nigroviridis* genomes were downloaded from Ensembl 68 [56]. We used BLASTN [57] to perform a search for the RAD-tag (and their paired-ends) against the 8 fish genomes. The parameters used were minimum alignment size 80 nt, minimum percentage of sequence identity 0.25 and maximum e-value 0.001 and low complexity mask on. All other parameters were set as default to account for the divergence and shortness for the sequences used. Sequences that aligned to more than one place in each genome were excluded from further analysis.

## Abbreviations

RAD: Restriction-site associated DNA; SNP: Single nucleotide polymorphism; QTL: Quantitative trait locus; MAS: Marker assisted selection; LG: Linkage group.

## Competing interests

The authors declare that they have no competing interests.

## Authors’ contributions

CP carried out linkage mapping and QTL analysis; MB analysed the sequence data to produce SNP genotype data, carried out synteny analysis and developed the combined marker sex prediction, MEC and AD carried out sex reversal and progeny testing of halibut, AD, MB and MO developed and carried out the SNP analyses, JBT and CP prepared RAD DNA libraries, KG was responsible for the RADtag sequencing, all authors contributed to writing and editing the manuscript, HM, DJP, JBT, AD and BJM designed and supervised the project. All authors read and approved the final manuscript.

## Supplementary Material

Additional file 1 **Samples origin and barcode.** Details each sample used: sample ID, family, gender, barcode used, number of extract raw reads and number of RAD-tags. Click here for file

Additional file 2 Genetic maps. Ordered markers: marker ID, linkage group and position (cM). Click here for file

Additional file 3 **Marker sequences and KASP assay primers.** List of the allele specific primers and common primer designed for the allele specific PCR genotyping assay of the 10 markers as well as their NCBI dbSNP accession numbers. Click here for file

Additional file 4 **Details of the KASP assay results.** Genotypes of the 94 assays. Click here for file

Additional file 5 **Syntenic map of the *H. hippoglossus *sex-associated region.** Each vertical block represents a segment of a different chromosome/scaffold; if the markers are on the same chromosome they are on the same block. All blocks are at the same scale. The dotted lines join the same markers from one species to the next, the solid lines are used to link markers between species further away. Regions of highest synteny have a blue background and have hyperlinks to Ensembl 68 genome browser. Click here for file
