# Supplementary figures and images for: Mapping the sex determination locus in the Atlantic halibut (Hippoglossus hippoglossus) using RAD sequencing
Source: BMC Genomics. 2013 Aug 20;14:566. doi: 10.1186/1471-2164-14-566 (PMC3765698; doi:10.1186/1471-2164-14-566)

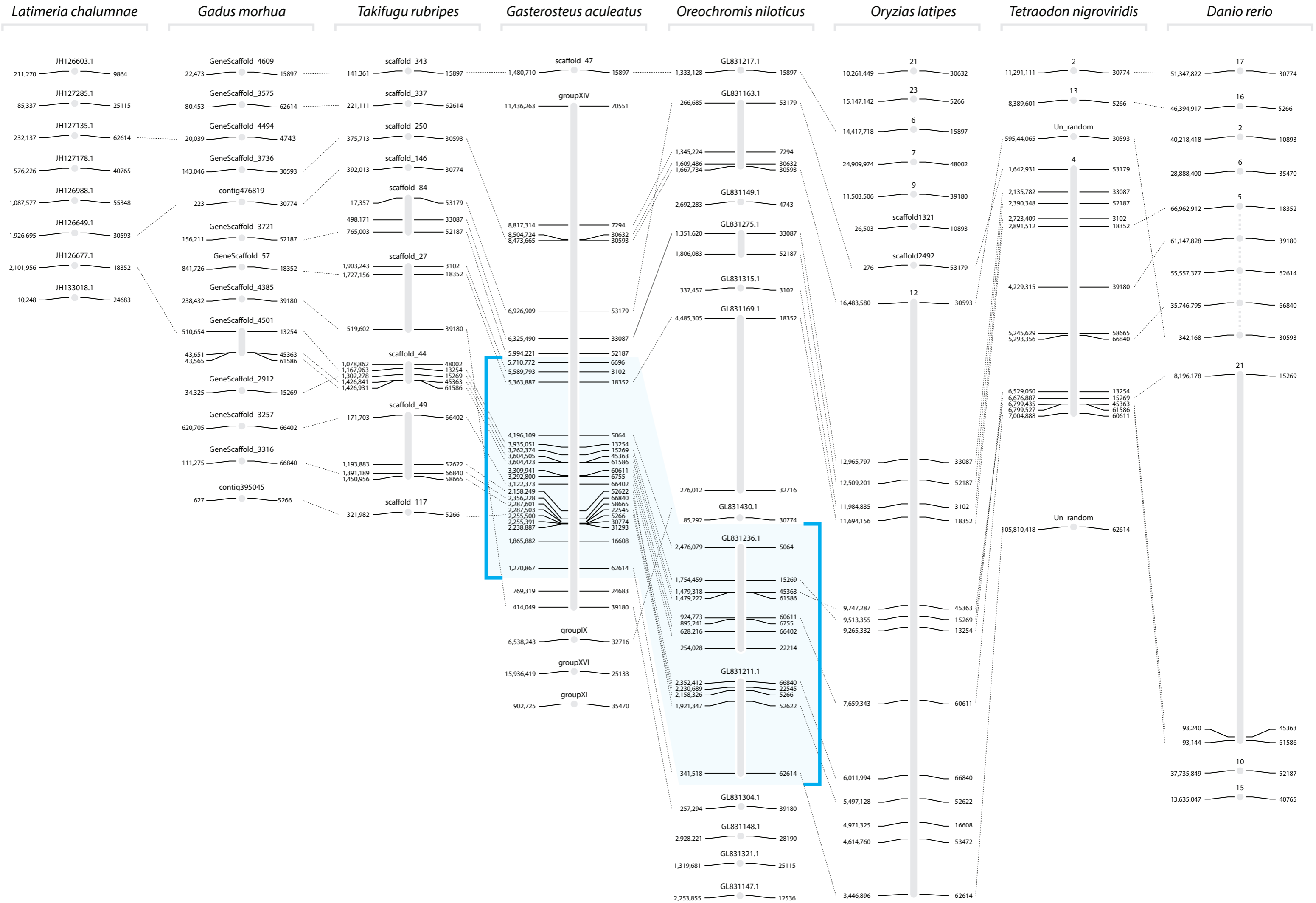

Supplement: Additional file 5 — Syntenic map of the H. hippoglossus sex-associated region. Each vertical block represents a segment of a different chromosome/scaffold; if the markers are on the same chromosome they are on the same block. All blocks are at the same scale. The dotted lines join the same markers from one species to the next, the solid lines are used to link markers between species further away. Regions of highest synteny have a blue background and have hyperlinks to Ensembl 68 genome browser. [file 1471-2164-14-566-S5.pdf]
